# Accurate MS-based Rab10 Phosphorylation Stoichiometry Determination as Readout for LRRK2 Activity in Parkinson's Disease

**DOI:** 10.1074/mcp.RA120.002055

**Published:** 2020-11-25

**Authors:** Özge Karayel, Francesca Tonelli, Sebastian Virreira Winter, Phillip E. Geyer, Ying Fan, Esther M. Sammler, Dario R. Alessi, Martin Steger, Matthias Mann

**Affiliations:** 1 Department of Proteomics and Signal Transduction, Max Planck Institute of Biochemistry, Martinsried, Germany; 2 Medical Research Council Protein Phosphorylation and Ubiquitylation Unit, University of Dundee, Dundee, United Kingdom; 3 Department of Neurology, School of Medicine, Ninewells Hospital, Ninewells Drive, Dundee, United Kingdom

**Keywords:** Biomarker: diagnostic, clinical proteomics, neurodegenerative diseases, parallel reaction monitoring, Parkinson disease, phosphorylation, protein kinases, targeted mass spectrometry, targeted therapies, selected ion monitoring

## Abstract

Pathogenic mutations in the Leucine-rich repeat kinase 2 (LRRK2) are the predominant genetic cause of Parkinson's disease (PD). They increase its activity, resulting in augmented Rab10-Thr73 phosphorylation and conversely, LRRK2 inhibition decreases pRab10 levels. Currently, there is no assay to quantify pRab10 levels for drug target engagement or patient stratification. To meet this challenge, we developed an high accuracy and sensitivity targeted mass spectrometry (MS)-based assay for determining Rab10-Thr73 phosphorylation stoichiometry in human samples. It uses synthetic stable isotope-labeled (SIL) analogues for both phosphorylated and nonphosphorylated tryptic peptides surrounding Rab10-Thr73 to directly derive the percentage of Rab10 phosphorylation from attomole amounts of the endogenous phosphopeptide. The SIL and the endogenous phosphopeptides are separately admitted into an Orbitrap analyzer with the appropriate injection times. We test the reproducibility of our assay by determining Rab10-Thr73 phosphorylation stoichiometry in neutrophils of LRRK2 mutation carriers before and after LRRK2 inhibition. Compared with healthy controls, the PD predisposing mutation carriers LRRK2 G2019S and VPS35 D620N display 1.9-fold and 3.7-fold increased pRab10 levels, respectively. Our generic MS-based assay further establishes the relevance of pRab10 as a prognostic PD marker and is a powerful tool for determining LRRK2 inhibitor efficacy and for stratifying PD patients for LRRK2 inhibitor treatment.

## Teaser

Pathogenic mutations in the Leucine-rich repeat kinase 2 (LRRK2) increase its activity leading to increased phosphorylation of Rab proteins. Here we introduce a sensitive and accurate assay to measure increased phospho Rab levels using synthetic stable isotope-labeled analogues for both phosphorylated and non-phosphorylated tryptic peptides surrounding Rab10-Thr73. Compared to healthy controls, carriers of mutated LRRK2 had 1.9-fold and those with VPS35 mutation 3.7-fold increased pRab10 levels in their neutrophils. Our generic MS-based assay helps to stratify PD patient and determine LRRK2 inhibitor efficiency in clinical trials.

## Graphical Abstract

**Figure:**
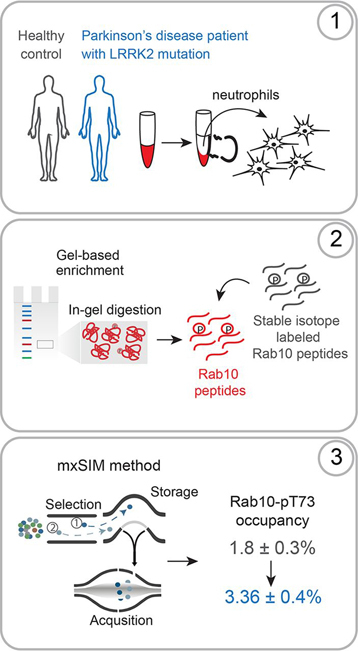

**Highlights**

• MS-based clinical assay that accurately determines phospho Rab10 occupancy.
• Stable isotope labeled phosphopeptide injected as a standard with endogenous tryptic phospho Rab peptide for accurate ratio determination.
• Determination of pRab levels in neutrophils of Parkinson disease patients.
• Relevance of pRab levels as marker of PD.

Parkinson's disease (PD) is the second most common neurodegenerative condition, and no disease-modifying therapies exist to date (1, 2). Although most PD cases are idiopathic, mutations in several genes have been linked to familial forms of the disease (3). Among those, mutations in the Leucine-rich repeat kinase 2 (LRRK2) comprise the predominant genetic cause of PD and account for 1% of sporadic and 4% of familial cases worldwide, and much higher in some populations (4). At least six pathogenic missense mutations in LRRK2, including the most frequent G2019S substitution, have been identified (5) and several studies confirmed that these mutations increase its kinase activity (5, 6, 7, 8). LRRK2-associated PD is clinically largely indistinguishable from idiopathic PD, suggesting that LRRK2 inhibition may be a useful a therapy for a larger group of patients (4). Clinical trials with selective LRRK2 kinase inhibitors are ongoing and have already passed phase 1.

Downstream targets of the LRRK2 kinase have long been enigmatic and controversial, but using genetic mouse models, specific inhibitors and a novel phosphoproteomics workflow, we have recently identified and verified a subset of Rab GTPases (Rab3A/B/C/D, Rab5A/B/C, Rab8A/B, Rab10, Rab12, Rab29, Rab35, and Rab43) as bona fide substrates (8, 9). Among these, Rab10 appears to be a key physiological kinase substrate (8, 9, 10, 11, 12, 13, 14). We found that LRRK2 directly phosphorylates Rab10 at Thr73 and all known pathogenic forms of LRRK2 enhance this phosphorylation (8, 9). Intriguingly, the PD-associated D620N mutation of the retromer complex protein VPS35 also activates LRRK2 kinase activity, which in turn results in augmented Rab10 phosphorylation (15). Thus, multiple PD-associated factors are interconnected and dysregulation of a common LRRK2-Rab signaling pathway appears to be an underlying cause of PD.

The LRRK2 autophosphorylation site Ser1292 has been widely used for assessing LRRK2 kinase activity (16, 17, 18, 19). However, its stoichiometry (percentage of molecules phosphorylated) appears to be extremely low and there is no sensitive phospho-specific antibody available to reliably detect and quantify phosphorylation at this site (6, 20, 21). We recently developed several high-affinity antibodies for detecting pRab proteins in cells and in tissues (14). Among those, a highly specific clone detects pThr73 levels in human peripheral blood cells of (mutant) LRRK2 G2019S and VPS35-D620N carriers with Parkinson's disease (11, 15). Although pRab10 levels were markedly increased in VPS35 D620N carriers, no statistically significant differences in pRab10 levels were detected by immunoblotting analysis when comparing controls to LRRK2-G2019S carriers in peripheral blood mononuclear cells (PBMCs) and neutrophils (11, 15). The reproducible quantification of immunoblots, particularly the detection of small (<2-fold) changes is challenging. In contrast, MS-based quantification has become a gold standard and has several advantages over traditional biochemical methods, as it is more specific and potentially more accurate. Importantly, MS allows simultaneous detection of both the phosphorylated peptide and the total protein pool and hence enables the direct calculation of the absolute fraction of the phosphorylated protein, also known as phosphorylation stoichiometry or occupancy (22).

There are several strategies differing in their accuracy, throughput, and applicability for measuring phosphorylation stoichiometry by MS (22, 23, 24, 25, 26, 27). One way is to compare the intensities of modified and unmodified peptides by label-free proteomics (27). However, because of potential differences in ionization efficiencies, the MS signals of individual peptides cannot be directly compared with each other. Stoichiometry determination based on heavy-to-light ratios of stable isotope-labeled (SIL) analogs of phosphorylated and nonphosphorylated proteolytic peptides and their endogenous counterparts can overcome this problem and allow a much more sensitive and precise readout for monitoring kinase activity (22, 23). Alternatively, a SIL recombinant protein that is chemically or enzymatically phosphorylated can also be used as the spike-in standard (28, 29). MS strategies determining stoichiometry that are combined with tailored targeted methods are especially suited for accurate quantification of low levels of a given phosphorylated analyte (30). Instruments with an Orbitrap mass analyzer can be operated in targeted scan modes such as high-resolution selected ion monitoring (SIM) or parallel reaction monitoring (PRM). In both methods, precursor ions are isolated with a narrow m/z range by a quadrupole mass filter before introduction into the Orbitrap analyzer, thereby providing increased signal to noise ratio for the ion of interest and allowing attomole-level limits of detection (31, 32). As no fragmentation is involved in SIM, quantification of selected ions relies on the high resolution in the Orbitrap mass analyzer alone. In contrast, in PRM, fragment ions are used for quantification of the peptide. Although that approach is more specific, the overall sensitivity of PRM can be lower, as the signal of a given precursor ion is distributed across multiple fragments (32).

Here we describe an accurate and highly-sensitive MS-assay for determining the Rab10-Thr73 phosphorylation stoichiometry and how it changes in Parkinson's disease. We evaluate it by comparing Rab10-Thr73 phosphorylation levels in healthy controls, idiopathic PD patients, and PD patients with defined genetic cause. Our assay enables the detection of subtle changes in Rab10 phosphorylation, which are beyond what is detectable by typical immunoassays. We show that pRab10 stoichiometry when measured precisely using our assay can serve as a robust target engagement and patient stratification marker in clinical studies.

## EXPERIMENTAL PROCEDURE

##### Experimental Design and Statistical Rationale

The experimental design and statistical rationale for each of the experiments conducted in this study are described in detail in each subsection and figure legend. Briefly, all neutrophil samples isolated from individuals were measured with technical replicates (*n* = 3). The LOQ experiment and IP experiments were also measured in technical replicates (*n* = 3). For CV experiments, we repeated MS measurements (analytical), and the workflow in the same gel (intra-assay) or in different gels (inter-assay) using the same phosphoprotein standard (*n* = 6).

##### Study Participants and Blood Sample Collection

Our assay was applied in the neutrophils collected from 14 healthy controls, three idiopathic PD patients, four LRRK2 G2019S and three VPS35 D620N mutation carriers with PD.

**Fig. 1:**
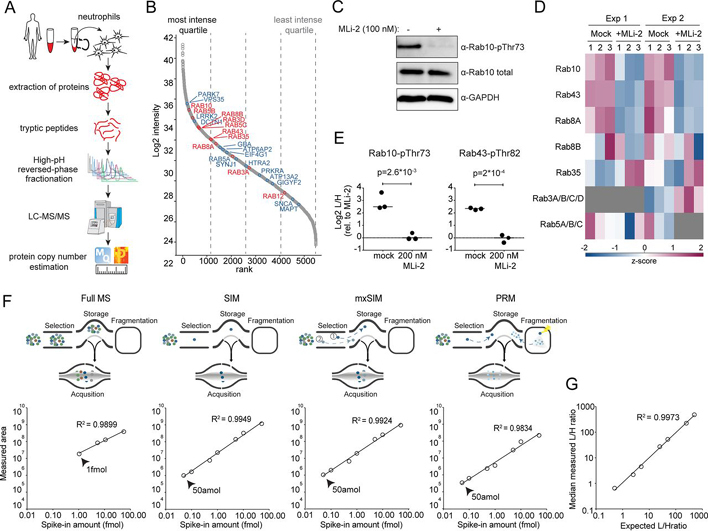
**Rab10-pThr73 as a readout for LRRK2 activity in human peripheral blood neutrophils.***A*, Workflow for global proteome analysis of human peripheral blood neutrophils. *B*, Proteins ranked according to their abundances across the global neutrophil proteome. Quartiles are indicated with dashed lines. LRRK2 phosphorylated Rab proteins and other PD-associated proteins are highlighted in red and blue, respectively. *C*, Western blot analysis of neutrophils (−/+ 200 nm MLi-2, 30 min) using anti-MJFF-pRAB10 (pThr73), Rab10 total and GAPDH antibodies. *D*, Heat map of z-scored phosphopeptide intensities of LRRK2-phosphorylated Rab proteins from pRab immunoprecipitation of neutrophil lysates (−/+ 200 nm MLi-2, 30 min, 3 technical replicates, *n* = 2). Missing values are in grey. *E*, Targeted MS-quantified Rab10-pThr73 and Rab43-pThr82 peptide intensities in immunoprecipitations of individual Rab10 and Rab43 proteins from neutrophils (−/+ 200 nm MLi-2, 30 min). *P*-values were assessed using unpaired Student's *t* test analysis. *F*, Limit of detection (LOD) of SIL Rab10-pThr73 tryptic peptide (FHpTITTSYYR) with various acquisition methods; full MS, SIM, mxSIM and PRM. Linear ranges of the dilution curves were assessed with singlets analyses of the SIL Rab10-pThr73 phosphopeptide (10 amol to 50 fmol, spiked-in a digest of HeLa). *G*, Limit of quantification (LOQ) for the Rab10-pThr73 tryptic peptide measured in mxSIM mode. 25 fmol of light phosphopeptide was mixed with variable amounts of its SIL counterpart (10 amol to 50 fmol) and spiked into a background of HeLa digest (3 technical replicates). Median ratios extracted from Skyline were plotted against the expected ratios.

**Fig. 2:**
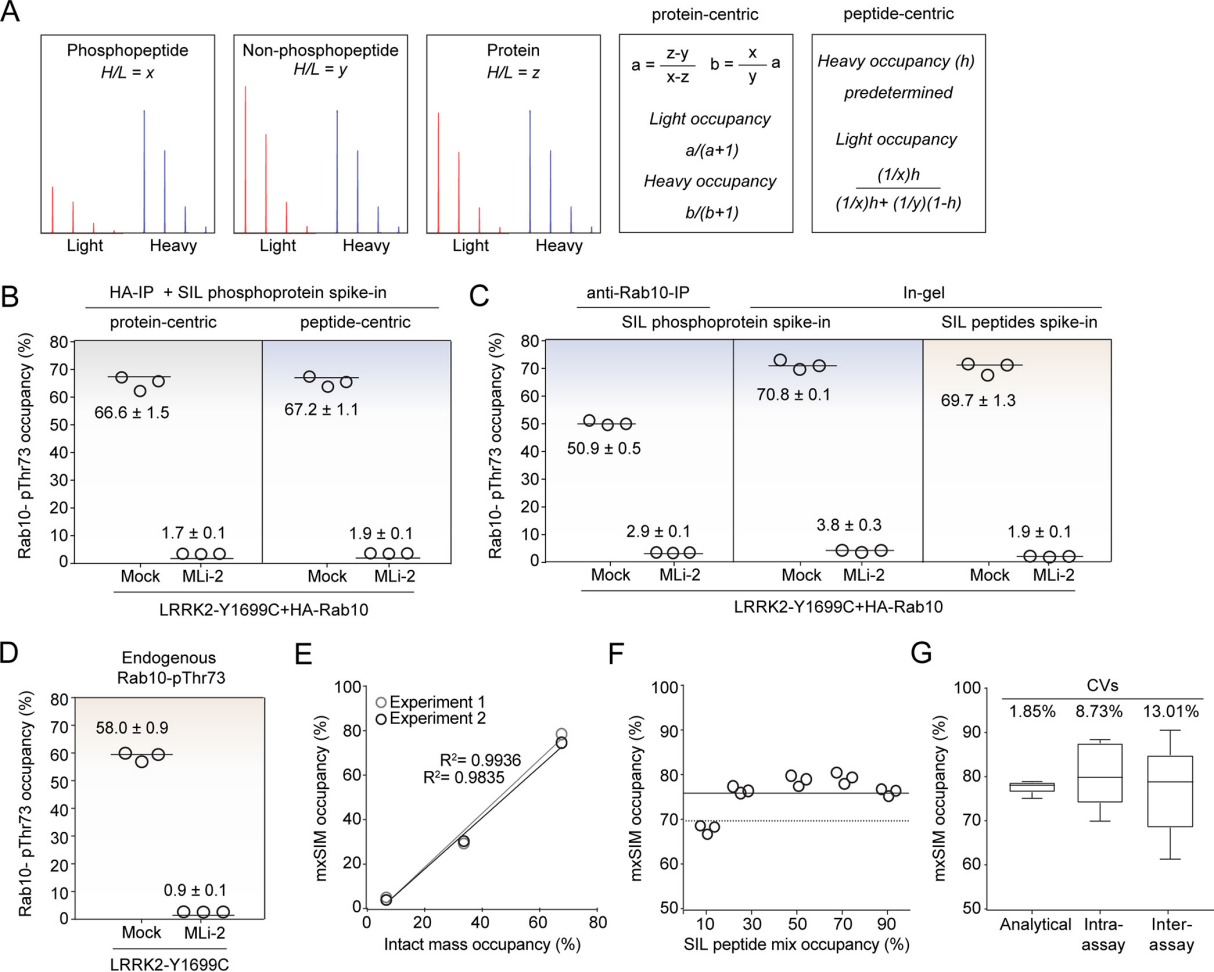
**mxSIM accurately determines Rab10-Thr73 phosphorylation stoichiometry.***A*, Heavy-to-light ratios and the formulas used for protein- and peptide-centric approaches. *B*, HA-Rab10-pThr73 occupancy in LRRK2-Y1699C-expressing HEK293 cells was determined using the protein- *versus* peptide-centric approaches after HA-IP. *C*, HA-Rab10-pThr73 occupancy determined using the peptide-centric approach with either SIL phosphoprotein or SIL peptide standard spike-in after enrichment by anti-Rab10-IP or SDS-PAGE followed by in-gel digestion in HEK293 cells expressing LRRK2-Y1699C (−/+ 200 nm MLi-2, 60 min). *D*, Endogenous Rab10-pThr73 occupancy determined using SIL peptide standards in mock and LRRK2-Y1699C expressing HEK293 cells (−/+ 200 nm MLi-2, 60 min). Samples from (*B*), (*C*), and (*D*) were analyzed in triplicates using the mxSIM method and the phosphorylation occupancies are presented as means ± SEM. *E*, Benchmarking our method using Thr73-phosphorylated unlabeled recombinant Rab10 proteins (1-175 aa) as standards. Correlation of the median occupancies determined either by intact mass analysis or by mxSIM in triplicates for two independent experiments. *F*, SIL phosphorylated and nonphosphorylated Rab10 peptides mixed to mimic 10, 30, 50, 70, and 90% occupancies and spiked into the digested recombinant pRab10 protein were measured using our mxSIM method in triplicates. The *solid line* represents the median occupancy (75.4 ± 1.5%) whereas the *dashed line* shows the estimated phosphorylation occupancy of the standard protein (70%) by intact mass analysis. *G*, CVs were calculated by repeating MS measurements (analytical), the workflow in the same gel (intra-assay) or in different gels (inter-assay) using the same phosphoprotein standard (*n* = 6).

**Fig. 3:**
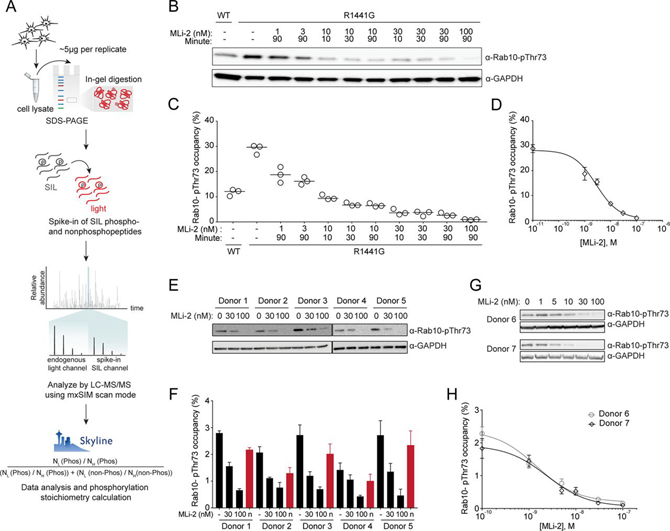
**Reliable determination of Rab10-pThr73 occupancy in cell lysates.***A*, Workflow for the Rab10-pThr73 occupancy assay. *B*, Immunoblotting of WT and R1441G knock-in MEFs treated with the indicated concentrations of MLi-2 for different intervals using monoclonal MJFF-pRAB10 (pThr73) and GAPDH antibodies. *C*, Rab10-pThr73 occupancies were determined by mxSIM in the same lysates (*n* = 3). *D*, Dose–response curve of Rab10-pThr73 occupancy in R1441G knock-in MEFs to generate occupancy based-IC_50_ values for MLi-2. Each data point represents the median of triplicate measurements in the samples treated with MLi-2 for 90 min. *E*, Immunoblotting of neutrophils isolated from healthy individuals (DMSO or 30 and 100 nm MLi-2 treated) using monoclonal MJFF-pRAB10 (pThr73) and GAPDH antibodies. *F*, Rab10-pThr73 occupancies were determined by mxSIM in the same lysates. Normalized Rab10- pThr73 occupancies by subtracting the MLi-2 treated values are shown in red. *G*, Immunoblotting of the neutrophils isolated from healthy individuals and treated with the indicated concentrations of MLi-2 using monoclonal MJFF-pRAB10 (pThr73) and GAPDH antibodies. *H*, Individual-specific dose–response curves to generate occupancy based-IC_50_ for MLi-2. Error bars represent SEM.

For setting up and validating our assay, we recruited seven volunteers from the Department of Proteomics and Signal Transduction at the Max Planck Institute of Biochemistry who kindly donated blood for our study. The data shown in Fig. 1, Fig. 2, Fig. 3 and supplemental Figs. S1–S3 are derived blood samples from healthy donors, which provided a written informed consent, with prior approval of the ethics committee of the Max Planck Society.

**Fig. 4:**
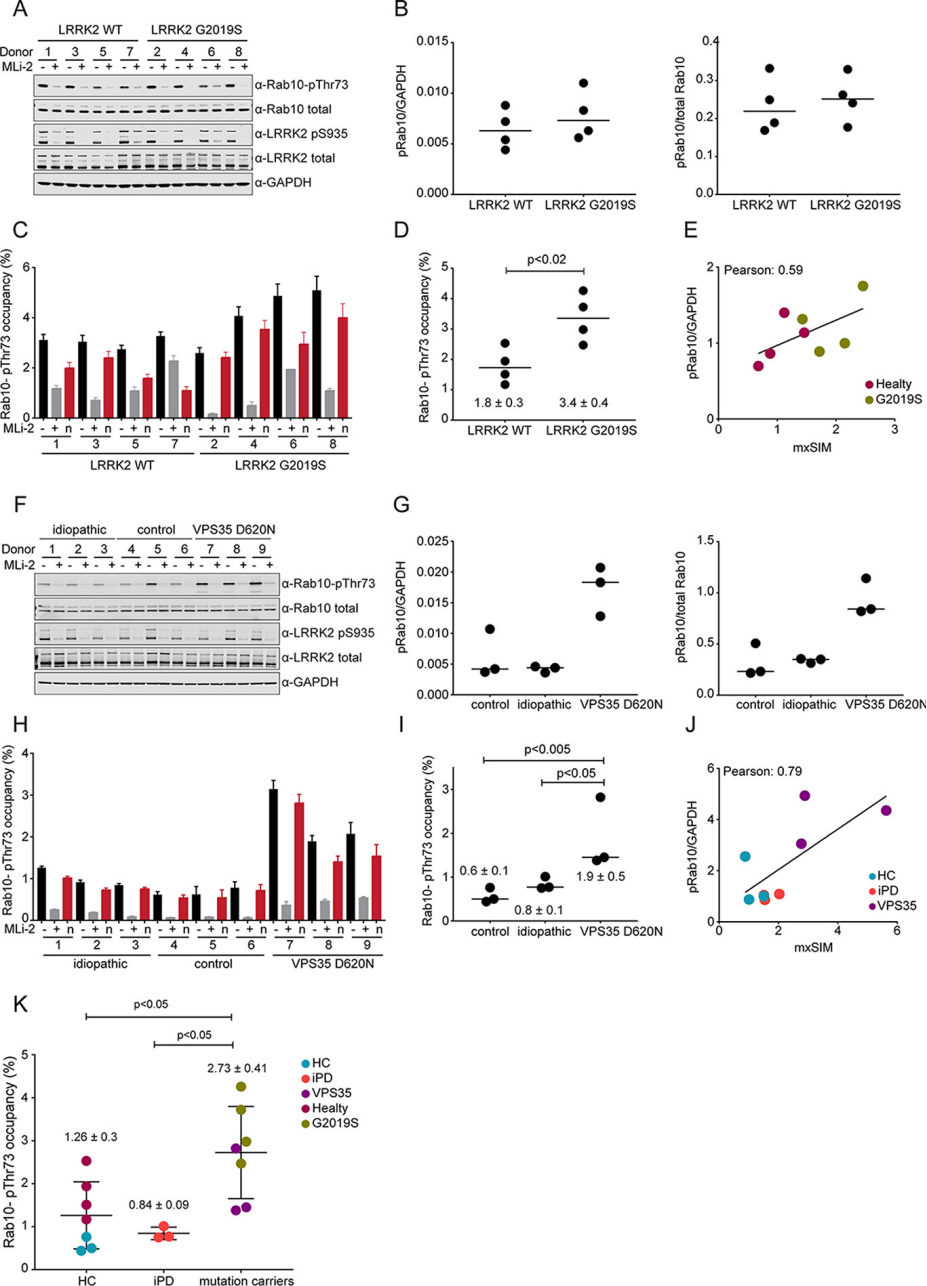
**Rab10-pThr73 occupancy in PD patient-derived neutrophils.***A*, Immunoblotting of neutrophils isolated from four PD patients with the G2019S LRRK2 mutation and four healthy controls (−/+100 nm MLi-2, 30 min) using anti total LRRK2, pSer935 LRRK2, total Rab10, MJFF-pRAB10 (pThr73) and GAPDH antibodies. *B*, Quantitation of immunoblots by analyzing phospho-Thr73 Rab10/total Rab10 ratio (*right*) and phospho-Thr73 Rab10/GAPDH ratio (left panel). *C*, Blind application of our mxSIM assay to the same mock (*black*) and MLi2-treated (grey) lysates (*n* = 3). Normalized Rab10- pThr73 occupancies by subtracting the MLi-2 treated values are also shown (*red*). Error bars represent SEM. *D*, Quantification of Rab10-pThr73 occupancy in controls and PD patients with the G2019S LRRK2 mutation. Each data point represents the median of triplicate measurements of untreated samples that are normalized to MLi2. One-way ANOVA with Bonferroni's multiple comparisons test was applied and the occupancies are presented as means ± SEM. *E*, Pearson correlation between immunoblotting and mxSIM assay. Plotted are fold changes of normalized Rab10-pThr73 levels (occupancies in mxSIM) relative to control cases. *F*, Immunoblotting of neutrophils isolated from controls, idiopathic PD patients and heterogeneous VPS35 D620N mutation carriers (−/+ 200 nm MLi-2, 30 min) using anti-total LRRK2, pSer935 LRRK2, total Rab10, MJFF-pRAB10 (pThr73) and GAPDH antibodies. *G*, Quantitation of immunoblots by analyzing phospho-Thr73 Rab10/total Rab10 ratio (*right*) and phospho-Thr73 Rab10/GAPDH ratio (*left* panel). *H*, Blind application of our assay to the same mock (*black*) and MLi2-treated (grey) lysates (*n* = 3). Normalized Rab10- pThr73 occupancies by subtracting the MLi-2 treated values are shown in red. Error bars represent SEM. *I*, Quantification of Rab10-pThr73 occupancy in controls, idiopathic PD and PD patients with heterogeneous VPS35 D620N mutation. The significant analysis was done as in (*D*). *J*, Same as (*E*) with healty controls (HC), idiopathic PD (iPD) and VPS35 D620N (VPS35). *K*, Quantification of Rab10-pThr73 occupancy between controls, idiopathic cases and PD patients with a defined mutation. The significant analysis was done as in (*D*).

For the experiments shown in Fig. 4, neutrophil lysates derived from either idiopathic PD patients, PD patients carrying a heterozygous LRRK2 G2019S or VPS35 D620N mutation or nonPD controls were used that had been previously used for publication ((11) and (15), respectively, supplemental Table S4). All procedures were performed in compliance with the local ethics review boards and all participants provided informed consent. These lysates were subjected to MS analysis in a blinded experimental set-up, with the identity of the lysates only being revealed after completion of the MS analysis.

##### Reagents

MLi-2 was purchased from Tocris Bioscience (cat# 5756). DIFP, HA-agarose and trypsin were from Sigma and LysC was from Wako. Microcystin-LR was from Enzo Life Sciences. Complete protease and phosphatase inhibitor tablets were from Roche. SIL peptides in absolute quantities (AQUA) were purchased from Sigma-Aldrich.

##### Antibodies

Anti-GAPDH (#5174), anti-HA (#3724), and anti-Rab10 (#8127) were from Cell Signaling Technologies. Rabbit mAb for total LRRK2 (UDD3, ab133518), Rab10 (T73) antibody [MJF-R21] and polyclonal phospho-Rab (initially raised against Rab8) antibody [MJF-R20] that efficiently immunoprecipitated multiple LRRK2 phosphorylated Rab proteins were purchased from Abcam (Burlingame, California). They were custom-made by Abcam in collaboration with the Michael J. Fox Foundation (Burlingame, California) (14, 33).

##### Plasmids

HA-Rab10 (DU44250) and Flag-LRRK2-Y1699C (DU13165). Full datasheets and reagents are available on https://mrcppureagents.dundee.ac.uk/.

##### Stable Isotope Labeling and Purification of the Rab10 Protein Standard

Buffer A: 50 mm HEPES pH 8.0, 500 mm LiCl, 1 mm MgCl_2_, 100 μm GDP, 1 mm TCEP and Elution buffer: Buffer A + 500 mm imidazole.

To obtain unlabeled Rab10 standard, human His-tagged Rab10 (residues 1-175) construct from Rai *et al.* (34) was expressed in *E. coli* BL21 (DE3) harboring the GroEL/S plasmid and protein expression was induced with isopropyl β-d-1-thiogalactopyranoside overnight. Cells were lysed by sonication in a buffer containing 50 mm HEPES pH 8.0, 500 mm LiCl, 1 mm MgCl_2_, 100 μm GDP, 1 mm TCEP (Buffer A) supplemented with several protease inhibitors including 30 μm Antipain and 50 μm Chymostain. Proteins were purified by Ni-NTA affinity chromatography. Briefly, bound proteins eluted with an imidazole gradient (25 mm-500mM) and imidazole was removed by washing with 500 μm ATP containing Buffer A. Further purification was done by ion-exchange chromatography (Q-Sepharose) followed by size exclusion chromatography using a Superdex 200 column. Peak fractions containing recombinant protein were pooled. Identity and purity of the standard protein were assessed by Maldi-TOF MS and SDS-PAGE. To obtain labeled Rab10 standard, we used an auxotrophic expression strain for arginine and lysine (29, 35). Cultures were grown in PA5052 minimal autoinduction media containing heavy Arg10 and Lys8 and cells were harvested for purification of SIL Rab10 protein standard as described earlier.

##### In Vitro LRRK2 Kinase Assays

Recombinant LRRK2 G2019S (Invitrogen, PV4881) and Rab proteins were incubated at 30°C for 30 min in kinase assay buffer (100 mm Tris–HCl, pH 7.5, 50 mm MgCl_2_, 5 mm EGTA, 1 mm GDP, 10 mm DTT, 25 mm β-glycerol phosphate, 5 mm Sodium orthovanadate, and 50 μm ATP). The reaction was terminated by addition of HG-10-102-0.

##### Cell Culture and Transfection

HEK293 and MEFs (WT and LRRK2-R1441G) cells were cultured in Dulbecco's modified Eagle's medium (Glutamax, Invitrogen) supplemented with 10% fetal calf serum, 100 U/ml penicillin and 100 μg/ml streptomycin. Transient transfections were performed 48 h before cell lysis using polyethylenimine PEI (Polysciences). Transfected cells were subjected to DMSO or MLi-2 (dissolved in DMSO) treatments at concentrations and periods of time as indicated in each figure legend. All cells were tested for mycoplasma contamination and overexpressing lines were verified by western blot analysis.

##### Neutrophil Isolation, Characterization, Treatments and Lysis

The procedure for neutrophil collection was done as described in detail in our video article (36). Pipetting of human blood were undertaken in a biological safety cabinet. Briefly, 10 ml of blood was collected into a blood collection tube and mixed gently by inverting tubes. Next, it was transferred into a 50 ml conical tube and mixed gently with 100 μl of EDTA Stock Solution (100 mm EDTA in PBS (PBS)). Neutrophils were isolated by immune-magnetic negative isolation using the MACSxpress® Neutrophil Isolation Kit (Miltenyi Biotec, Cat# 130-104-434). For 8 ml of blood, one vial of ‘Isolation Cocktail’ (magnetic beads) from the neutrophil isolation kit, delivered as a lyophilized pellet, was reconstituted by adding 0.25 ml of Buffer A and 0.25 ml of Buffer B in that order. The mixture was mixed by gently pipetting and added to 8 ml of whole blood in 15 ml falcon tube. The blood sample containing the mixture was gently mixed by inversion and incubated at room temperature for 5 min. The falcon tube was next placed into the magnetic field of the MACSxpress Separator (# 130-098-308) for 15 min. The magnetically labeled cells (non-neutrophils) adhere to the wall of the tube whereas the aggregated erythrocytes sediment to the bottom. The supernatant (∼ 7 ml) containing the enriched neutrophils was carefully pipetted into a new 15 ml falcon tube, avoiding touching the magnetic beads attached to the sides of the falcon tube as well as the red blood cells at the bottom of the tube. The supernatant containing the isolated neutrophils was centrifuged at 300 × *g* for 10 min at room temperature (acceleration and deceleration is both 5 using a Beckman Coulter Allegra X-15R Centrifuge). To ensure the removal of erythrocytes, the pelleted neutrophil cells were resuspended in 10 ml of 1 × Red Blood Cell Lysis Solution (# 130-094-183), incubated for 10 min at room temperature and centrifuged at 300 × *g* for 10 min at room temperature. To assess the purity and recovery of the enriched neutrophils an aliquot was taken at this stage and we later fluorescently stained cells with CD14-PerCP, CD15-PE, CD16-APC, and CD193-FITC and analyzed by flow cytometry. Cell debris, dead cells were excluded from the analysis based on scatter signals and DAPI. Finally, the cell pellet was resuspended with 10 ml room temperature RPMI 1640 media by gentle pipetting. At this stage, purified cells were subjected to MLi-2 (dissolved in DMSO) treatment at concentrations and periods of time as indicated in each figure legend. An equivalent volume of DMSO was added to negative control samples. Following treatment, cells were pelleted through centrifugation at 500 g for 5 min. Cells were then resuspended in 5 ml of ice-cold PBS and centrifuged again at 500 g for 5 min. The supernatant was carefully removed by pipetting. Cells were lysed in 300 μl of ice-cold Nonidet P-40 buffer (50 mm Tris–HCl, pH 7.5, 1% (v/v) Triton X-100, 1 mm EGTA, 1 mm sodium orthovanadate, 50 mm NaF, 0.1% (v/v) 2-mercaptoethanol, 10 mm 2-glycerophosphate, 5 mm sodium pyrophosphate, 0.1 μg/ml microcystin-LR, 270 mm sucrose, 0.5 mm DIFP (Sigma, Cat# D0879) supplemented with protease and phosphatase inhibitors (Roche)). Lysate was clarified by centrifugation at 16,000 rpm for 15 min at 4 °C after a liquid nitrogen freeze-thaw cycle. Protein concentration was measured using Bradford assay (Thermo Scientific), snap-frozen and stored at −80 °C. The whole procedure from the collection of blood to the freezing of neutrophil lysates was up to 4 h.

##### Immunoblot Analysis

Cell lysates were mixed with 4 × SDS–PAGE loading buffer [250 mm Tris–HCl, pH 6.8, 8% (w/v) SDS, 40% (v/v) glycerol, 0.02% (w/v) Bromphenol Blue and 4% (v/v) 2-mercaptoethanol] to final total protein concentration of 1 μg/μl and heated at 85 °C for 10 min. Samples were loaded onto NuPAGE Bis-Tris 4–12% gel (Invitrogen) and electrophoresed at 180 V for 1 h with MOPS SDS running buffer followed by transfer onto the nitrocellulose membrane (GE Healthcare, Amersham Pharmacia Biotech Protran Supported 0.45 μm NC) at 100 V for 90 min on ice in the transfer buffer (48 mm Tris–HCl and 39 mm glycine). Membrane was then cropped into pieces: from top of the membrane to 75 kDa to incubate with rabbit anti-LRRK2 UDD3 antibody, from 75 to 30 kDa to incubate with rabbit anti-GAPDH antibody and from 30 kDa to the bottom of the membrane to incubate with rabbit monoclonal antibodies for anti-Rab10-pThr73 or anti-Rab10. Antibodies diluted in 5% (w/v) BSA in TBS-T to a final concentration of 1 μg/ml. All blots were incubated in primary antibody overnight at 4 °C. Before secondary antibody incubation, membranes were washed three times with TBS-T (20 mm Tris/HCl, pH 7.5, 150 mm NaCl and 0.2% (v/v) Tween 20) for 10 min each. Membranes were incubated with secondary antibody multiplexed with goat anti-rabbit diluted in 5% (w/v) nonfat dry milk (NFDM) in TBS-T (1:5000 dilution) for 1 h at room temperature. They were next washed with TBS-T three times for 10 min. Protein bands were detected using an ECL solution (Amersham Pharmacia Biotech ECL Western Blotting Detection Reagents (GE Healthcare)) and the ImageQuant LAS 4000 imaging system. For the immunoblots shown in Fig. 4*A* and 4*F*, the membranes were developed using the LI-COR Odyssey CLx Western Blot imaging system.

##### Cells Lysis and Pull-Downs

Cells were lysed in either Nonidet P-40 buffer (50 mm Tris-HCl, pH 7.5, 120 mm NaCl, 1 mm EDTA, 6 mm EGTA, 20 mm NaF, 15 mm sodium pyrophosphate and 1% Nonidet P-40 supplemented with protease and phosphatase inhibitors (Roche)) or Triton X-100 buffer (50 mm Tris–HCl, pH 7.5, 1% (v/v) Triton X-100, 1 mm EGTA, 1 mm sodium orthovanadate, 50 mm NaF, 0.1% (v/v) 2-mercaptoethanol, 10 mm 2-glycerophosphate, 5 mm sodium pyrophosphate, 0.1 μg/ml microcystin-LR, 270 mm sucrose, 0.5 mm DIFP (Sigma, Cat# D0879) supplemented with protease and phosphatase inhibitors (Roche)). Lysates were clarified by centrifugation at 16,000 rpm for 15 min at 4 °C after a liquid nitrogen freeze-thaw cycle. Protein concentrations were measured using Bradford assay (Thermo Scientific), snap-frozen and stored at −80 °C. For HA pulldowns, lysates were incubated with HA-agarose resin for 2 h (25 μl resin per 100 μg of lysates). For immunoprecipitation using total Rab10 or phospho-Rab, lysates were incubated with antibodies in manufacturer's recommend dilutions or concentrations overnight at 4 °C and subsequently incubated with 25 μl of Protein-A/G-agarose beads for 2 h at 4 °C. To remove unspecific binders, beads were washed twice with matching lysis buffer and twice with 50 mm Tris-HCl (pH 7.5). SIL phosphorylated Rab10 protein was mixed with the beads at this step if used as standrad. Washes were followed by on-bead digestion overnight at 37 °C with trypsin (∼500 ng/pulldown in urea buffer [2M urea dissolved in 50 mm ammonium bicarbonate] or SDC buffer [1% (w/v) SDC in 100 mm Tris-HCL pH 8.5]). The resulting peptides were processed as described in ‘LC–MS/MS sample preparation’.

##### In-Gel Digestion Protocol

Cell lysates were mixed with 4 × SDS/PAGE sample buffer (250 mm Tris/HCl, pH 6.8, 8% (w/v) SDS, 40% (v/v) glycerol, 0.02% (w/v) Bromphenol Blue and 4% (v/v) 2-mercaptoethanol) and heated at 85 °C for 5 min. SIL Rab10 phosphoprotein was mixed with samples at this step if used as standard. 20-30 μg sample (5 μg per MS analysis) was loaded onto NuPAGE Bis-Tris 4-12% gel (Invitrogen) and electrophoresed at 180 V. After SDS-PAGE, gel was washed once with deionized water and stained with 0.1% Coomassie Blue R250 in 10% acetic acid, 40% methanol and 60% deionized water for 20 min and subsequently destained by soaking for at least 2 h in 10% acetic acid, 40% methanol, and 60% deionized water with at least two changes of the solvent (until the background is nearly clear). Gel band corresponding to 20-30 kDa is excised and chopped into smaller pieces (∼ 1 × 1 mm) and placed in clean 1.5 ml tubes. Gel pieces are washed two or three times with 50% 50 mm ABC/50% EtOH for 20 min at RT and then completely dehydrated by incubating for 10 min in absolute EtOH. Samples were dried in a speed-vac for 10 min (45 °C) until the gel pieces were bouncing in the tube. Gel pieces were rehydrated in 300 μl of 1% (w/v) SDC buffer (10 mm TCEP, 40 mm CAA, trypsin in 100 mm Tris-HCL pH 8.5) per sample and placed at 37 °C overnight. The next day, 300 μl of isopropanol buffer (1% TFA in isopropanol) was added and shaken for 10 min and spin down. The liquid was transferred into a fresh tube. Further 200 μl of isopropanol buffer was added to the gel pieces and shaken vigorously for 20 min at RT. It was combined with that from the previous step. Samples were directly loaded onto SDB-RPS stage tips and processed as described in ‘LC–MS/MS sample preparation’.

##### Human Neutrophil Proteome Digestion, in-StageTip Purification and Fractionation

The neutrophil cell pellet was prepared with the iST Kit for proteomic sample preparation (P.O. 00001, PreOmics GmbH). In brief, this involved denaturation, alkylation, digestion and peptide purification. Reduction of disulfide bridges, cysteine alkylation and protein denaturation was performed at 95 °C for 10 min. After a 5 min cooling step at room temperature, trypsin and LysC were added to the mixture at a ratio of 1:100 μg of enzyme to micrograms of protein. Digestion was performed at 37 °C for 1 h. 20 μg of peptides was loaded on two 14-gauge SDB-RPS StageTip plugs. Samples were directly loaded onto SDB-RPS StageTips processed as described in ‘LC–MS/MS sample preparation’. Clean peptides were separated using the high-pH reversed-phase ‘Spider fractionator’ into 24 fractions as described previously to generate deep proteomes (37).

##### LC–MS/MS Sample Preparation

StageTips (38) were prepared by inserting two 16-gauge layers of a SDB-RPS matrix (Empore) into a 200 μl pipette tip using an in-house prepared syringe device as described previously (39). The StageTips were centrifuged using an in-house 3D-printed StageTip centrifugal device at 1500 g. The acidified peptides (1% TFA v/v) were loaded onto the StageTips that were later washed with 1% TFA in isopropanol and subsequently 2% ACN/0.2% TFA. Elution was performed using 60 μl of 50% ACN/1.25% NH_4_OH or 80% ACN/1.25% NH_4_OH. Eluates were collected in PCR tubes and dried using a SpeedVac centrifuge (Eppendorf, Concentrator plus) at 60 °C. Peptides were resuspended in buffer A* (2% ACN/0.1% TFA) and briefly sonicated (Branson Ultrasonics) before LC–MS-MS analysis. To calculate absolute Rab10-pThr73 occupancy, we spiked SIL phosphorylated and nonphosphorylated counterpart peptides into samples at this step. Light contamination in heavy standards was not detected up to 100 fmol of spike-in amount (the highest amount tested). Given that the detection limits of these peptides with our mxSIM assay were below 50 amol, light contamination is determined to be <0.05%.

##### LC–MS/MS Measurements

Peptides were loaded on a 20 or 50 cm reversed phase column (75 μm inner diameter, packed in house with ReproSil-Pur C18-AQ 1.9 μm resin (Dr. Maisch GmbH)). Column temperature was maintained at 60 °C using a homemade column oven. An EASY-nLC 1200 system (Thermo Fisher Scientific) was directly coupled online with the mass spectrometer (Q Exactive HF-X, Thermo Fisher Scientific) via a nano-electrospray source, and peptides were separated with a binary buffer system of buffer A (0.1% formic acid (FA)) and buffer B (80% acetonitrile plus 0.1% FA), at a flow rate of 300 nl/min. Peptides were eluted with a 45 min gradient of 5–60% buffer B (0.1% (v/v) FA, 80% (v/v) ACN). After each gradient, the column was washed with 95% buffer B for 5 min.

The mass spectrometer was programed to acquire in targeted scan mode in which every full scan with resolution 60,000 at 200 *m*/*z* (3 × 10^6^ ions accumulated with a maximum injection time of 20 ms) was followed by two multiplexed selected ion monitoring (mxSIM) scans employing multiplexing degree of two to record both light (endogenous) and heavy counterpart simultaneously for either phosphorylated or nonphosphorylated Rab10-pT73 tryptic peptides. Each SIM scan covered a range of *m*/*z* 150–2000 with resolution 120,000 (10^5^ ions accumulated with a maximum injection time of 230 ms for both light and heavy counterparts, 1.4 *m*/*z* isolation window and 0.4 m/z isolation offset). *m*/*z* values of doubly-charged Rab10-pT73 tryptic (FHpTITTSYYR) light and heavy peptides (arginine labeled (13C, 15N)) were defined as follows: 684.8028 and 689.8070 whereas doubly-charged Rab10 nonphosphorylated tryptic (FHTITTSYYR) light and heavy peptides (arginine labeled (13C, 15N)) were targeted at *m*/*z* of 644.8197 and 649.8238.

For the LOD experiment (Fig. 1), the mass spectrometer was programed to acquire in either full scan mode alone or SIM or PRM combined with a full scan. Full scan acquisition was performed with a resolution of 120,000 at 200 *m*/*z* (3 × 10^6^ ions accumulated with a maximum injection time of 230 ms) to cover the scan range of 350–1650 *m*/*z*. SIM acquisition was performed using a resolution of 120,000 at 200 *m*/*z*, isolation windows of 1.4 *m*/*z* with 0.4 m/z offset, target AGC values of 2 × 10^5^, and a maximum injection time of 230 ms. PRM acquisition was performed using a resolution of 60,000 at 200 *m*/*z*, isolation windows of 1.4 *m*/*z* with 0.4 *m*/*z* offset, target AGC values of 2 × 10^5^, and a maximum injection time of 130 ms. Fragmentation was performed with a normalized collision energy of 27.

The neutrophil proteomes were analyzed using an LC–MS instrumentation consisting of an EASY-nLC 1200 system (Thermo Fisher Scientific) combined with a Q Exactive HF Orbitrap (Thermo Fisher Scientific) and a nano-electrospray ion source (Thermo Fisher Scientific). The purified peptides were separated on a 50 cm HPLC column (75 μm inner diameter, in-house packed into the tip with ReproSil-Pur C18-AQ 1.9 μm resin (Dr. Maisch GmbH)). Of each of the 24 fractions around 0.5 μg peptides were analyzed with a 45 min gradient. Peptides were loaded in buffer A (0.1% FA, 5% DMSO (v/v)) and eluted with a linear 35 min gradient of 3–30% of buffer B (0.1% FA, 5% DMSO, 80% (v/v) ACN), followed by a 7 min increase to 75% of buffer B and a 1 min increase to 98% of buffer B, and a 2 min wash of 98% buffer B at a flow rate of 450 nl/min. Column temperature was kept at 60 °C by a Peltier element containing in-house developed oven. MS data were acquired with a Top15 data-dependent MS/MS scan method (topN method). Target values for the full scan MS spectra was 3 × 10^6^ charges in the 300–1650 *m*/*z* range with a maximum injection time of 55 ms and a resolution of 120,000 at *m*/*z* 200. Fragmentation of precursor ions was performed by higher-energy C-trap dissociation (HCD) with a normalized collision energy of 27. MS/MS scans were performed at a resolution of 15,000 at *m*/*z* 200 with an ion target value of 5 × 104 and a maximum injection time of 25 ms.

For intact mass analysis, proteins were loaded on a reversed-phase column (Phenomenex AerisTM 3.6 μm Widepore C4 100 mm × 2.1 mm inner diameter, 200 Å pore size). An Agilent 1100 HPLC system was coupled online with the mass spectrometer (microTOF, Bruker Daltonik) and masses were recorded from 800–3000 *m*/*z*. Proteins were separated with a binary buffer system of buffer A (0.05% TFA in H_2_O, pH 2.0) and buffer B (0.05% TFA in can, pH 2.0) at a flow rate of 250 nl/min. Proteins were eluted with a gradient of 20–80% buffer B in 20 min. After each gradient, the column was washed with 95% buffer B for 1 min and 20% buffer B for 3 min. Data were processed using the CompassTM ‘DataAnalysis’ software from Bruker Daltonik, deconvoluted with ‘MaximumEntropy’ and an instrument resolving power of 10,000.

##### Data Analysis and Development of mxSIM Assay to Calculate Phosphorylation Stoichiometry

The type of our targeted occupancy assay is a Tier 2, according to previously published guidelines (40). For the calculation of absolute Rab10-pThr73 occupancies, raw MS data were processed using Skyline 4.2 (0.19072) (41), which is an open source software project and can be freely installed. Raw files were directly imported into Skyline in their native file format. After data import, graphical displays of chromatographic traces were manually inspected for proper peak picking of MS1 filtered endogenous peptides based on co-eluting stable isotope-labeled peptides. All quantitation performed for phosphorylation occupancy calculations in this study were done on the precursor ion level. Only the most abundant first two peaks of the isotope cluster were used for quantitation. Peptide areas (AUCs) for the nonphosphorylated tryptic Rab10 peptide (FHTITTSYYR, *m*/*z* 644.8197++) and the phosphorylated tryptic Rab10 peptide (FHpTITTSYYR, *m*/*z* 684.8028++) with their *r* = 13C615N4 heavy analogues were extracted to derive light-to-heavy ratios. The absolute quantification was determined by comparing the abundance of the known SIL internal standard peptides with the native peptides. The phosphorylation stoichiometry/occupancy is calculated by taking the ratio of the total amount of phosphorylated fraction to the total amount of both phosphorylated and nonphosphorylated forms, which is always represented as percentage (%). When using mxPRM method, the median ratio of all unique y ions that belong to the phosphorylated and nonphosphorylated tryptic Rab10 peptides was used to calculate occupancy. All details for occupancy calculations in this study are provided in the supplemental Table S3.

For the deep proteome of human neutrophils, raw MS data were processed using MaxQuant version 1.5.6.8 (41, 42) against the Human UniProt FASTA database containing 21,052 entries (UniProt, release 2017) and a list of 245 potential contaminants. Enzyme specificity was set to trypsin, and the search included cysteine carbamidomethylation as a fixed modification and N-acetylation of protein and oxidation of methionine as variable modifications. Up to two missed cleavages were allowed for protease digestion, and peptides had to be fully tryptic. MaxQuant uses individual mass tolerances for each peptide, whereas the initial maximum precursor mass tolerances were set to 20 ppm in the first search and 4.5 ppm in the main search, and the fragment mass tolerance was set to 20 ppm. The false discovery rate was controlled with a target-decoy approach at less than 1% for peptide spectrum matches and less than 1% for protein group identifications.

Bioinformatic analyses in this study were performed with Perseus (www.perseus-framework.org) (43), Microsoft Excel and data visualized using GraphPad Prism (GraphPad Software) or RStudio (https://www.rstudio.com/).

## RESULTS

##### Rab10-pThr73 Serves as a Readout for LRRK2 Activity in Human Peripheral Blood Neutrophils

We decided to monitor Rab phosphorylation in in human peripheral blood neutrophils, as these cells can be sampled in a minimally invasive way. To explore which Rab GTPases were expressed in these cells and which of them could serve as a readout for LRRK2 activity in a quantitative MS-based assay, we first isolated neutrophils from whole blood using a negative selection approach. We assessed the recovery and the purity of the enriched cells by flow cytometry and found it to be more than 98%, with > 96% of them viable (supplemental Fig. S1). Proteomic analysis resulted in 5,488 quantified proteins, for which we estimated the copy numbers per cell using the proteomic ruler approach (45) (Fig. 1*A* and supplemental Table S1). Encouragingly, LRRK2, the vesicular protein VPS35 and many of the Rab proteins that are LRRK2 substrates were in the highest quartile of the abundance-ranked proteome (Fig. 1*B*). The bona-fide LRRK2 substrate Rab10 had the highest copy number among all detected Rab proteins with an estimated 1,820,000 copies per cell, suggesting its suitability as a marker for LRRK2 activity in PD (supplemental Fig. S2).

To determine whether Rab10 is the only Rab family member that is phosphorylated by LRRK2 in neutrophils, we first treated freshly isolated cells with the selective LRRK2 inhibitor MLi-2 and confirmed the down-regulation of Rab10-pThr73 by immunoblotting (11) (Fig. 1*C*). In the same lysates, we enriched phosphorylated Rab proteins with the previously described pThr-specific Rab antibody and subjected the eluates to LC–MS/MS analysis (9). We identified 19 Rab GTPases in total, of which 7 were previously shown to be LRRK2 targets (9) (Fig. 1*D*, supplemental Fig. S3, supplemental Table S2). In two independent pThr-specific Rab antibody pulldown experiments, Rab10, Rab43 and Rab8a phospho-protein levels decreased significantly upon MLi-2 treatment, indicating that these Rabs are targeted by LRRK2 in this system. To directly confirm LRRK2-mediated phosphorylation of these proteins, we immunoprecipitated them and quantified Rab10-pThr73, Rab43-pThr82 and Rab8a-pThr72 phosphopeptides by targeted MS (Selected Ion Monitoring, SIM, see Experimental procedure). Only Rab10 and Rab43 phosphorylation sites were down-regulated more than 4-fold after LRRK2 inhibitor treatment, demonstrating that the phosphorylation status of these Rabs can be used as readout for LRRK2 kinase activity (Fig. 1*E*).

The high abundance of Rab10 in neutrophils and the promise of pThr73 as a biomarker for Parkinson's disease encouraged us to develop a highly accurate and sensitive targeted MS-based assay for quantifying phosphorylated Rab10 in human cells at the peptide level. To maximize sensitivity, we explored a multiplexed SIM (mxSIM) setup on a linear quadrupole Orbitrap instrument (Q Exactive HF-X) (46). In our strategy, the SIL analog of the phosphorylated tryptic Rab10-Thr73 peptide acts as a sentinel peptide, as it can be spiked-in in high amounts and elutes simultaneously with its endogenous light counterpart. Light (endogenous) as well as SIL counterpart phosphopeptides are consecutively isolated by narrow quadrupole isolation windows but simultaneously injected into the Orbitrap mass analyzer. We set the maximum total ion accumulation time to 230 ms, but allocate 90% of this to the endogenous phosphopeptide, thus boosting its signal and increasing the sensitivity of our assay many-fold.

To compare the sensitivity and the accuracy of (mx)SIM with regular full-MS scanning and with PRM, we mixed variable amounts of the SIL Rab10 phosphopeptide (10 amol to 50 fmol) with 50 ng of a tryptic Hela digest and measured the SIL pRab10 peptide intensity using our different scan protocols. Relative to full-MS scanning, in which the entire mass range (300–1650 *m*/*z*) is analyzed, SIM, either multiplexed or not, provided a 20-fold increase in sensitivity with a limit of detection (LOD) of 50 amol (Fig. 1*F*). PRM performed equally well in terms of sensitivity, however, SIM had a somewhat higher quantification accuracy (R^2^ of 0.992 *versus* 0.983). For this reason, and because it was sufficiently specific in our system, we decided to develop a Rab10-pThr73 quantification assay based on mxSIM.

To determine the maximum heavy-to-light ratio and the limit of quantification (LOQ) of our method, we mixed 25 fmol of the light pRab10 peptide with variable amounts of its heavy counterpart (10 amol to 50 fmol) in 50 ng of HeLa digest. The results for mxSIM indicate excellent reproducibility of quantification (R^2^ = 0.997) (Fig. 1*G*). Because of the differential filling strategy, we accurately quantified heavy-to-light ratios of Rab10 phosphopeptides of up to 1:500 (25 fmol light and 50 amol heavy Rab10 peptide).

##### mxSIM Precisely and Accurately Determines Rab10-Thr73 Phosphorylation Stoichiometry

To evaluate protein and peptide-centric approaches for determining Rab10-Thr73 phosphorylation stoichiometry, we expressed and purified Rab10 (1-175 aa) from an auxotroph *E.coli* strain, which allowed for incorporation of SIL lysine and arginine into the newly synthesized protein (29, 35). We were able to phosphorylate 50% of the recombinant protein by LRRK2, as shown by intact mass analysis, and bottom-up proteomics confirmed Thr73 as the phosphorylation site (supplemental Fig. S4A and S4B). This is a very suitable proportion as both phosphorylated and nonphosphorylated peptides are needed as standards and we therefore decided to use this SIL recombinant phosphoprotein for quantifying the percentage of pRab10 in cells. For this purpose, we immunoprecipitated HA-Rab10 from LRRK2-Y1699C-expressing HEK293 cells, either treated with MLi-2 or not, and mixed the enriched protein with our SIL standard before joint tryptic digestion (supplemental Fig. S5A–S5B). We then derived the Rab10-Thr73 phosphorylation stoichiometry, as described above, to be 66.6 ± 1.5%. Subsequently, we used the same measurement but calculated the occupancy with the peptide-centric approach, yielding 67.2 ± 1.1% occupancy. As both approaches gave nearly identical results, we decided to use the simpler peptide centric approach for all further calculations (Fig. 2*B*).

Detection of sub-stoichiometric, post-translationally modified peptides in complex mixtures by proteomics is challenging, even with sensitive targeted methods, and requires one or more upfront enrichment steps. To select a suitable antibody for enriching Rab10, we compared one recognizing the total protein with one recognizing the HA-epitope tag in HA-Rab10 expressing cells. Unexpectedly, the Rab10 directed antibody yielded significantly lower apparent pThr73 occupancy as compared with the antibody directed against the epitope tag (50.9 ± 0.5% *versus* 67.2 ± 1.1%). An likely explanation for this discrepancy could be that the anti-Rab10 antibody preferentially recognizes the nonphosphorylated fraction of the total protein pool (Fig. 2*B* and 2*C*). To address our challenge with a different approach that does not rely on antibodies, we separated the cell lysate mixed with the SIL phosphoprotein standard on SDS-PAGE, excised the region of ∼15-30 kDa and digested the proteins using trypsin, followed by mxSIM analysis. This resulted in a measured Rab10-pThr73 occupancy of 70.8 ± 1.0%, in almost perfect agreement with the occupancy obtained by the anti-HA immunoprecipitation approach (Fig. 2*B* and 2*C*). Finally, we used SIL phosphorylated and nonphosphorylated peptides for deriving the Rab10-Thr73 phosphosite occupancy and again found that 69.7 ± 1.3% of the protein was phosphorylated in the same cell lysate; again an almost identical occupancy value as the one obtained from spike-in of the SIL phosphoprotein (Fig. 2*C*). We therefore decided to combine gel-separation, SIL peptide spike-in and the peptide-centric calculation method. With these tools in hand, we extended our in-gel digestion workflow to endogenous Rab10-pThr73 occupancy determination. We found that 58.0 ± 0.9% of the protein was phosphorylated in LRRK2-Y1699C-transfected cells, whereas the treatment with MLi-2 almost completely abolished the phospho-occupancy (0.89 ± 0.03%) (Fig. 2*D*).

To further benchmark our assay, we incubated recombinant Rab10 (1-175 aa) with LRRK2 and stopped the phosphorylation reaction by adding the LRRK2 inhibitor HG-10-102-0 at defined time intervals. Next, we determined the percentages of the phosphorylated Rab10 proteins by intact MS and compared these values to the occupancies obtained using our mxSIM method (supplemental Fig. S6A–S6D). This experiment revealed an excellent correlation between these methods in two independent experiments with R^2^ of 0.984 and 0.994 (Fig. 2*E*). To specifically test the SIL peptide spike-in part of the approach, we mixed SIL phosphorylated and nonphosphorylated Rab10 peptides in 1:9, 1:2.3, 1:1, 2.3:1, and 9:1 ratios to mimic phosphosite occupancies of 10, 30, 50, 70, and 90%, respectively, and mixed them with our recombinant Rab10 phosphoprotein. We then derived the Rab10-pThr73 occupancy based on the heavy-to-light ratios of both phosphorylated and nonphosphorylated peptides (Fig. 2*F*). The calculated mean occupancy for our recombinant Rab10 phosphoprotein was 75.3 ± 1.5%, for mxSIM and 70% for intact mass analysis. We also measured the Rab10-pThr73 occupancy of the same recombinant protein and obtained 77.4 ± 0.4% for PRM and 77.6 ± 0.6% for mxSIM (supplemental Fig. S7A). Finally, we determined the analytical, the intra- and the inter-assay variabilities, which yielded excellent coefficients of variations (CVs) of 1.9%, 8.7% and 13.0%, respectively (Fig. 2*G*). This demonstrates that our mxSIM assay is highly accurate and reproducible.

##### In-Gel Digestion Combined with mxSIM Can Detect Subtle Changes in Rab10-Thr73 Phosphorylation within Cells

Most pathogenic LRRK2 mutations, including R1441C/G/H, increase LRRK2 kinase activity and significantly stimulate Rab10 protein phosphorylation in mouse and human cells and tissues (6, 7, 8, 9). To determine whether our assay was sufficiently accurate and robust to detect small differences of LRRK2 activity in cells, we treated WT and LRRK2 R1441G knock-in mouse embryonic fibroblasts (MEFs) with increasing concentrations of MLi-2 and determined Rab10-Thr73 phosphorylation occupancies (Fig. 3*A*). In parallel, we controlled LRRK2 inhibitor efficacy by immunoblotting and probing for Rab10-pThr73 (Fig. 3*B*). Compared with WT, in which the Rab10-pThr73 occupancy was 12.1 ± 0.6%, we found a 2.45-fold increase in R1441G (29.7 ± 0.9%) (Fig. 3*C*). Rab10-pThr73 occupancy already decreased by 1.5-fold (18.8 ± 1.5%) upon treatment with 1 nm of MLi-2 and by almost 2-fold (15.4 ± 0.7%) with 3 nm of the inhibitor (Fig. 3*B*–3*C*). The corresponding Rab10-pThr73 immunoblot signals also showed some decrease, however, reliable and precise quantification of these bands was difficult.

We reasoned that our data should also allow determination Rab10-pThr73 IC_50_ of MLi-2 directly from the occupancies. We extracted a value of 3 nm, well within the 3-10 nm range estimated by our previous phos-tag Rab10 analysis (Fig. 3*D*) (12). Together, our results establish that our assay can reliably detect small differences in phosphorylation occupancy in cultured cells.

We next addressed our main objective: to determine whether our assay is suitable and sufficiently sensitive for quantifying the percentage of Rab10-pThr73 in human peripheral blood. To this end, we isolated neutrophils from five healthy volunteers. Upon treatment with either 30 nm or 100 nm of MLi-2, we found a decrease in Rab10-pThr73 by immunoblotting (Fig. 3*E*). Judged by this, the level of phosphorylated Rab10 protein was low in all individuals. Applying our workflow in the manner described above, showed that the median occupancy was 2.3 ± 0.2% in DMSO-treated cells and that it decreased to 1.3 ± 0.1% after 30 nm and to 0.6 ± 0.1% after 100 nm MLi-2 treatment (Fig. 3*F*). These occupancies correspond to 225,500 ± 15,500 (DMSO), 115,270 ± 6190 (30 nm MLi-2) and 60,160 ± 4600 (100 nm MLi-2) phosphorylated Rab10 molecules per cells (supplemental Table S3) We independently confirmed these results by PRM (supplemental Fig. S7B).

Intriguingly, the residual Rab10-pThr73 signal after MLi-2 treatment suggested that a kinase other than LRRK2 acts on this site. This finding raises interesting questions about the disease mechanism but in our current context, it presents a source of variability. To account for this, we corrected the occupancies of each untreated sample by subtracting the corresponding baseline occupancy (after MLi-2-treatment). This better separated disease phenotypes in clinical cohorts (Fig. 3*F*). Finally, we measured a MLi-2 dose-response curve (1–100 nm) using neutrophils from two donors and determined their occupancy based-IC_50_ values. These were similar in both donors (2.4 nm and 3 nm), further demonstrating the high reproducibility of our method even *in vivo* (Fig. 3*G* and *H*).

##### PD Patients Have Increased Rab10-Thr73 Phosphorylation Stoichiometry

The frequency of the LRRK2 G2019S mutation is 1% in patients with sporadic PD and 4% in hereditary PD patients with up to 30–40% in certain populations such as Ashkenazi Jews and North African Berbers (4). To investigate the central question of whether our assay can stratify PD patients with elevated LRRK2 activity in clinical trials, we analyzed Rab10-pThr73 levels in neutrophils of four LRRK2-G2019S carriers with PD, together with an equal number of healthy controls in a blinded experimental setup (see Experimental Procedures, supplemental Table S4). Each neutrophil population was treated with DMSO or 100 nm MLi-2 and all samples were subjected to quantitative immunoblot analysis for Rab10-pThr73 levels, revealing a clear reduction in Rab10 phosphorylation upon LRRK2 inhibition (Fig. 4*A*). LRRK2-pSer935 levels were also monitored by immunoblot analysis in the lysates to confirm the efficacy of MLi-2 treatment. Total Rab10 levels did not differ between the various genotypes (Fig. 4*A*). We quantified Rab10 phosphorylation by normalizing it with either total Rab10 or GAPDH (Fig. 4*B*). Although the data show that Rab10-pThr73 levels are slightly increased in G2019S carriers when normalized with GAPDH, the observed differences did not reach statistical significance (Fig. 4*B*). The median occupancy of normalized Rab10-pThr73 was 1.8 ± 0.3% in healthy controls and increased by almost 2-fold (3.36 ± 0.4%) in G2019S carriers (Fig. 4*C*–4*D*). In MLi-2 treated samples, the occupancy decreased 5-fold on average for both groups. Despite a good correlation between the two measurements (*r* = 0.59), MS-based quantification was better at separating controls from G2019S carriers (*p* = 0.019) (Fig. 4*E*).

Our previous work demonstrated that VPS35 controled LRRK2 activity and that the D620N substitution resulted in increased LRRK2 activity as assessed by monitoring phospho-Rabs levels (15). To test if our assay can determine LRRK2 activity in PD patients with the VPS35 D620N mutation, we measured Rab10-Thr73 phosphorylation occupancies in neutrophils isolated from three heterozygous patients, as well as three age-matched idiopathic PD patients and three nonPD controls (see Experimental Procedure, supplemental Table S4). We treated the neutrophils from each subject with either DMSO or 200 nm MLi-2 before cell lysis. In accordance with our previous work, immunoblot analysis revealed significantly elevated Rab10-pThr73 levels in VPS35 D620N patients (Fig. 4*F*–4*G*). The median occupancies were 0.6 ± 0.1%, 0.8 ± 0.1% and 1.9 ± 0.5% in healthy controls, idiopathic and heterozygous VPS35 D620N patients, respectively, and decreased 6.9-fold on average in MLi-2 treated cells (Fig. 4*H*). Our MS-based assay also confirmed significantly higher levels of Rab10-pThr73 levels in VPS35 D620N patients compared with controls and idiopathic cases and we observed a very good correlation between the two measurements (*r* = 0.79) (Fig. 4*I*–4*J*). Compared with controls, there was 1.5-fold increase in Rab10-pThr73 levels in idiopathic PD with both assays, however, the coefficient of variation in the control group was 30% with the mxSIM occupancy assay and 51% by immunoblotting (Fig. 4*G*- and 4*I*).

In conclusion, we here determined pRab10 occupancies in neutrophils of 7 healthy controls and 7 PD patients with defined mutations (four LRRK2 G2019S and three VPS35 D620N). Compared with healthy controls, neutrophils of mutation carriers with PD robustly displayed a higher fraction of phosphorylated Rab10, namely a 2.2-fold increase in pRab10 levels (*p*-value <0.05) (Fig. 4*K*). These results demonstrate that our assay can accurately ascertain the LRRK2 kinase activity in human derived samples, including its increase in mutation carriers, and suggest that it can be applied to stratify patients.

## DISCUSSION

Here, we have established an accurate and highly-sensitive MS-based assay for determining the percentage of Rab10-Thr73 phosphorylation in samples collected from PD patients. Using stable-isotope labeled spike-in peptides and a differential filling strategy for an Orbitrap analyzer, our range of quantification extended down to 50 attomoles and we were able to capture phosphorylation differences as small as those characterize controls and mutation carriers (1.26% to 2.73 in our human neutrophil samples). We confirmed Rab10-Thr73 phosphorylation as a direct readout for LRRK2 activity and specifically applied our assay to monitor LRRK2 activity in human neutrophils. We found that these cells contain relatively high levels of both LRRK2 and Rab10 and that their isolation is straightforward, although rapid isolation with minimal protease activation is important (11). As a next step, it will be interesting to investigate whether pRab10 can be detected in postmortem brain tissues or bodily fluids such as cerebrospinal fluid (CSF) and urine. Large and readily accessible collections of these bio-fluids could be used to further establish pRab10 as a bona-fide biomarker for PD disease progression.

After careful evaluation of spike-in standard type (SIL phosphoprotein *versus* peptides) and occupancy calculation approach (protein- *versus* peptide-centric approach), we opted for the peptide-centric approach in conjunction with the use of SIL peptides standards. Although spike-in of a labeled protein has the advantage of automatically accounting for the digestion efficiency, addition of labeled peptides makes the occupancy determination more generally applicable, as labeled peptides can readily be obtained at very high purity.

Furthermore, our assay uses separation of Rab10 by SDS-PAGE, which is followed by in-gel digestion. This strategy substantially enriches Rab10 and allows quantifying Rab phosphorylation with high sensitivity. When applied to large cohorts, a limitation of our assay is its low throughput. To solve this, the in-gel step in our workflow should be eliminated and as alternative antibodies that recognize both GDP and GTP bound Rab10 could be employed. There are considerable efforts currently being invested into producing these tools 14. Alternatively, nonhydrolyzable GTP analogues could be used to enrich Rab proteins before MS analysis.

We determined pRab10 occupancy in neutrophils of 14 healthy controls, three idiopathic PD patients, four LRRK2 G2019S and three VPS35 D620N mutation carriers with PD. Despite the small number of patients and the large intragroup variability, the difference between control and PD cases with LRRK2 G2019S and VPS35 D620N mutations reached statistical significance. Analysis of larger cohorts and inclusion of patient samples with higher LRRK2 activity, such as the R1441G/C mutations, should add statistical power and further establish pRab10 occupancy as a bona-fide marker of LRRK2 activity in PD. It will be exciting to investigate whether PD patients with increased pRab10 levels would also benefit from LRRK2 inhibitor treatment.

We find that the levels of Rab10-Thr73 phosphorylation are stable but very low in human neutrophils. This is also true for PD-associated LRRK2-G2019S mutation carriers where activation of the LRRK2 kinase is modest whereas individuals with a VPS35 D620N mutation demonstrate much higher Rab10-Thr73 phosphorylation, reflecting its much greater catalytic effect on the LRRK2 kinase. Our results show that changes in pRab10 can be used as direct readout for LRRK2 activity and the very low percentage of pRab10 that we observe ex vivo may explain the age-dependent, incomplete penetrance of the LRRK2 G2019S mutation. It will be interesting to compare pRab10 percentages of matched disease-manifesting and nonmanifesting LRRK2 G2019S mutation carriers at younger and older ages.

Our results could explain how minimal changes in the total pool of a phosphorylated protein could trigger pathogenesis. Stratification for Rab10-pThr73 levels in idiopathic and mutation carrying PD patients may identify individuals with increased LRRK2 kinase activity who would most likely benefit from LRRK2 kinase inhibitor treatment. Furthermore, our mass spectrometric assay is completely generic and not restricted to a disease. It could be applied to study any other kinase-substrate relation in clinical research. For example, phosphosite occupancies of prominent oncogenic factors could give important information on how phosphorylation stoichiometry influences tumorigenesis (14).

In summary, our generic and sensitive MS-based assay accurately measures phosphorylation stoichiometry of substrates of interest. Presently, it can be used for ongoing clinical LRRK2 inhibitor studies, in which the target engagement, dosing and efficacy of the compounds needs to be evaluated, and we are currently pursuing this. We envision that our assay can also be applied to further investigate the role of LRRK2 in the PD, potentially revealing new upstream players of LRRK2 pathway, and determining whether they have a potential in treating PD.

## DATA AVAILABILITY

Proteomics raw data have been deposited to the ProteomeXchange Consortium via the PRIDE partner repository with the data set identifier PXD015219 and PXD019814 and at Panorama Web (https://panoramaweb.org/pRab10.url). Annotated spectra for the results can be viewed through MS-Viewer using the search key zbcwt6pszv (44).
