# Supplementary material for: Accurate MS-based Rab10 Phosphorylation Stoichiometry Determination as Readout for LRRK2 Activity in Parkinson's Disease
Source: Mol Cell Proteomics. 2020 Nov 25;19(9):1546–60. doi: 10.1074/mcp.RA120.002055 (PMC8143643; doi:10.1074/mcp.RA120.002055)
Supplement: Supplementary file 1 [file mmc1.zip › mmc1/159671_1_supp_550277_qc39fh.pdf]

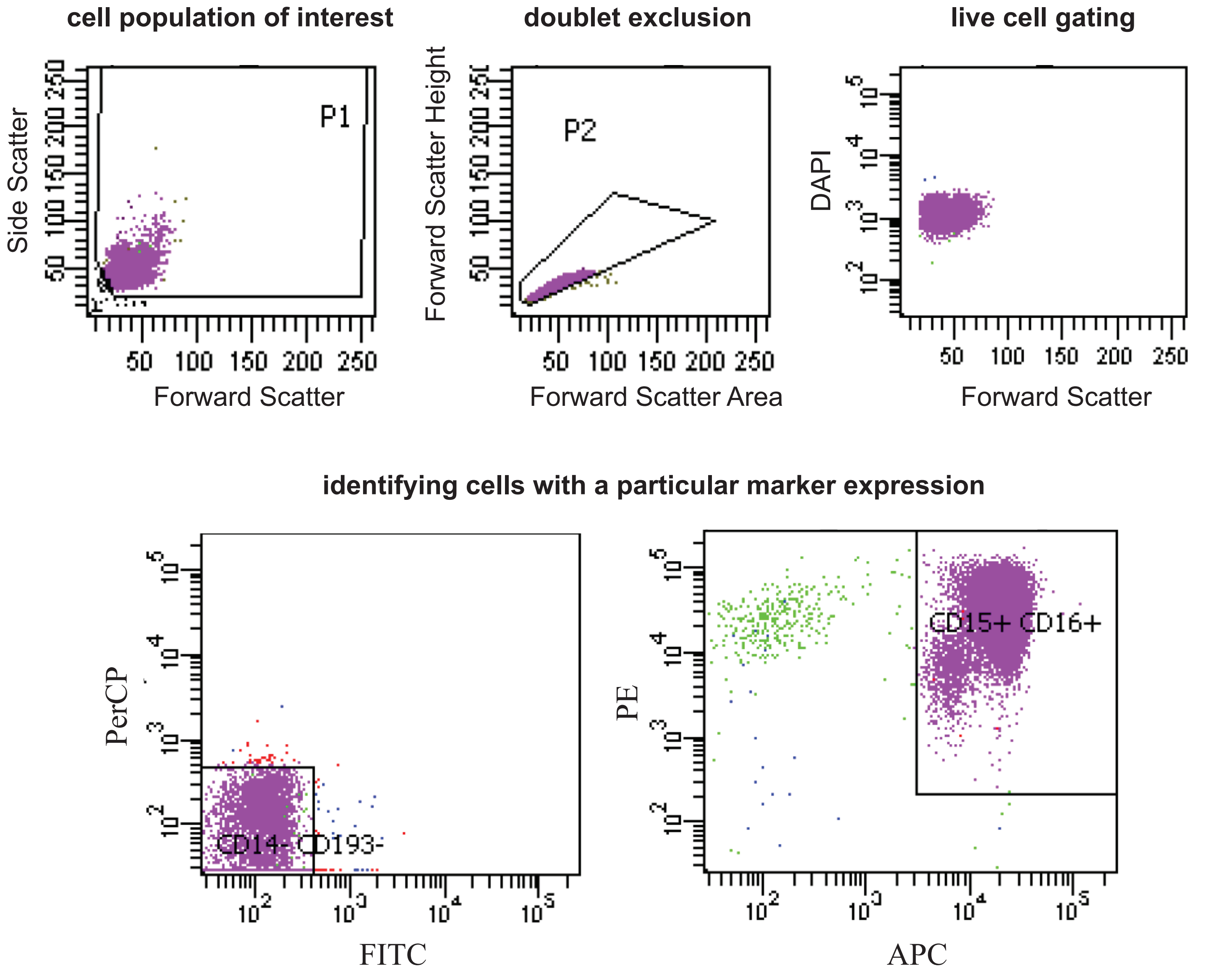

Supplementary Fig 1. A representative flow cytometry analysis determining the purity of the isolation method and viability of isolated neutrophils. Plots and population gating shown is for the donor used in the experiment in Figure 1C-D. Cell debris, non-leukocytes, and dead cells were excluded from the analysis based on scatter signals and DAPI. Purity was assessed by staining with CD14-PerCP (monocytes and macrophages), CD15-PE (granulocytes, monocytes, neutrophils and eosinophils), CD16-APC (neutrophils, macrophages and eosinophils), and CD193-FITC (eosinophils). The population which is CD15+ and CD16+ but CD14- and CD193- represents the neutrophils. The isolated cells had a viability >96% and a purity>98%.

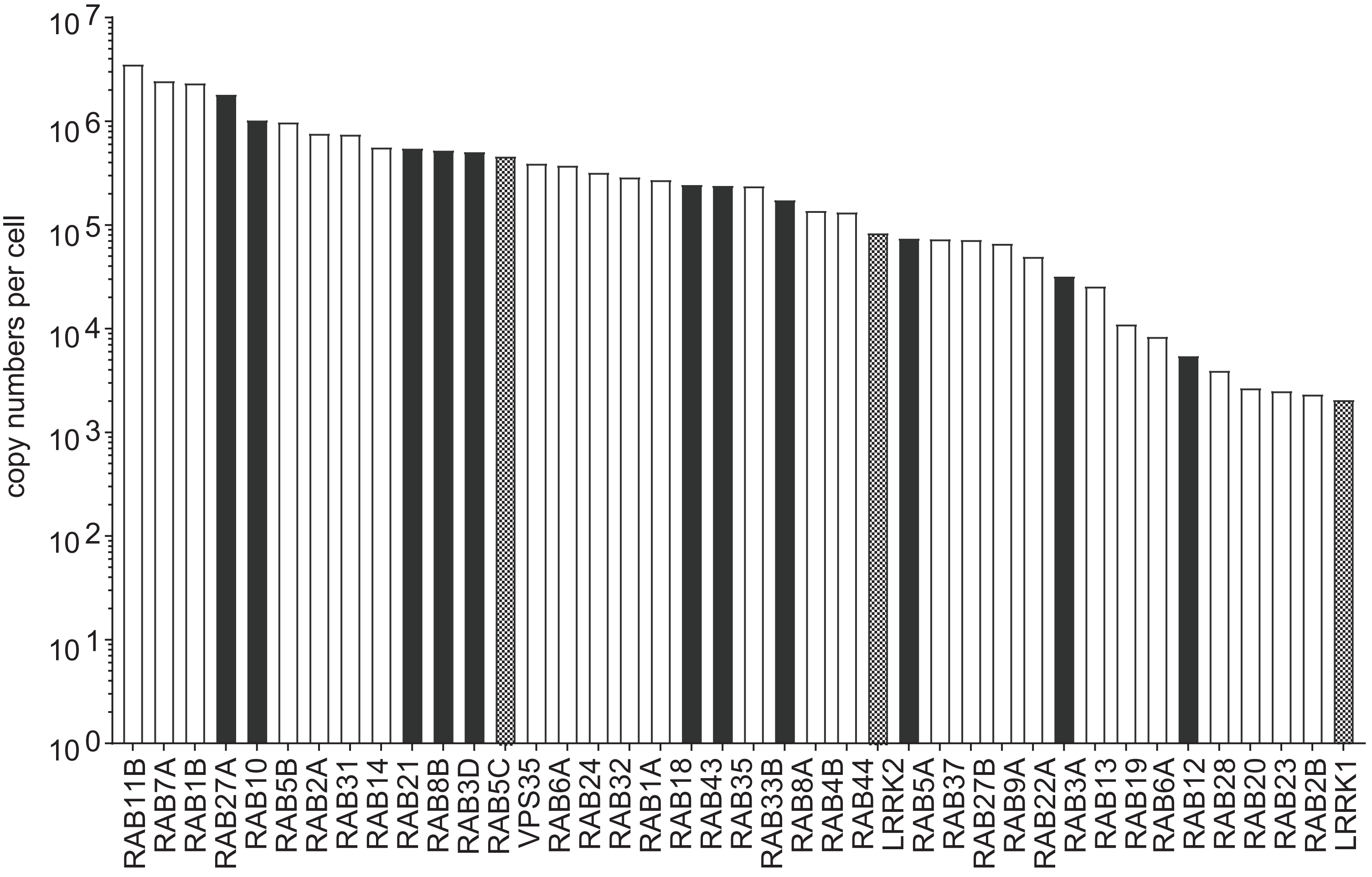

Supplementary Fig. 2. The copy numbers of the protein products of PD-associated genes, LRRK2 and VPS35, and Rab GTPases in a human neutrophil based on the cumulative histone amount considered proportional to the expected DNA amount per cell were estimated using the proteomic ruler approach (Wiśniewski et al., 2014), implemented in Perseus software (Tyanova et al., 2016). Black bars show the Rab proteins which were shown to be LRRK2 targets where as grey bars show LRRK1 and LRRK2 proteins.

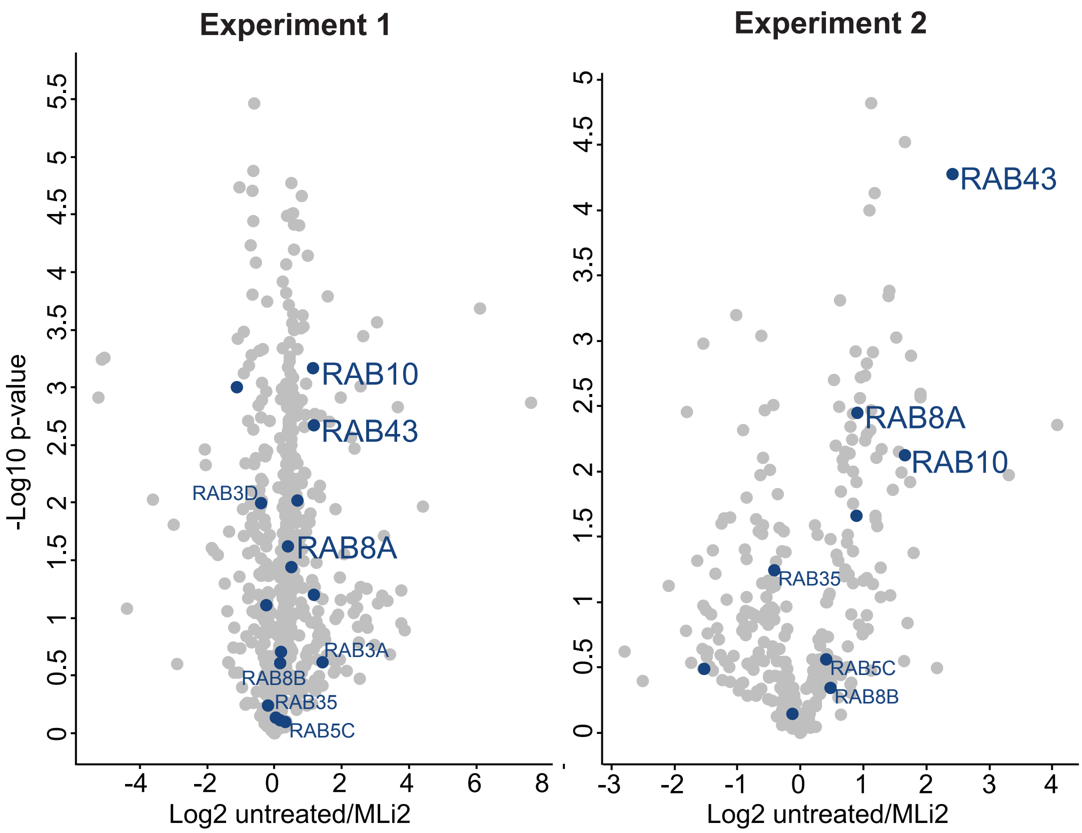

Supplementary Fig. 3. LRRK2 dependent Rab GTPase phosphorylations in human neutrophils. Volcano plots of the pRab immunoprecipitations (untreated:right and MLi-2-treated:left). All Rab proteins identified were highligted in blue and LRRK2 substrates were also labeled.

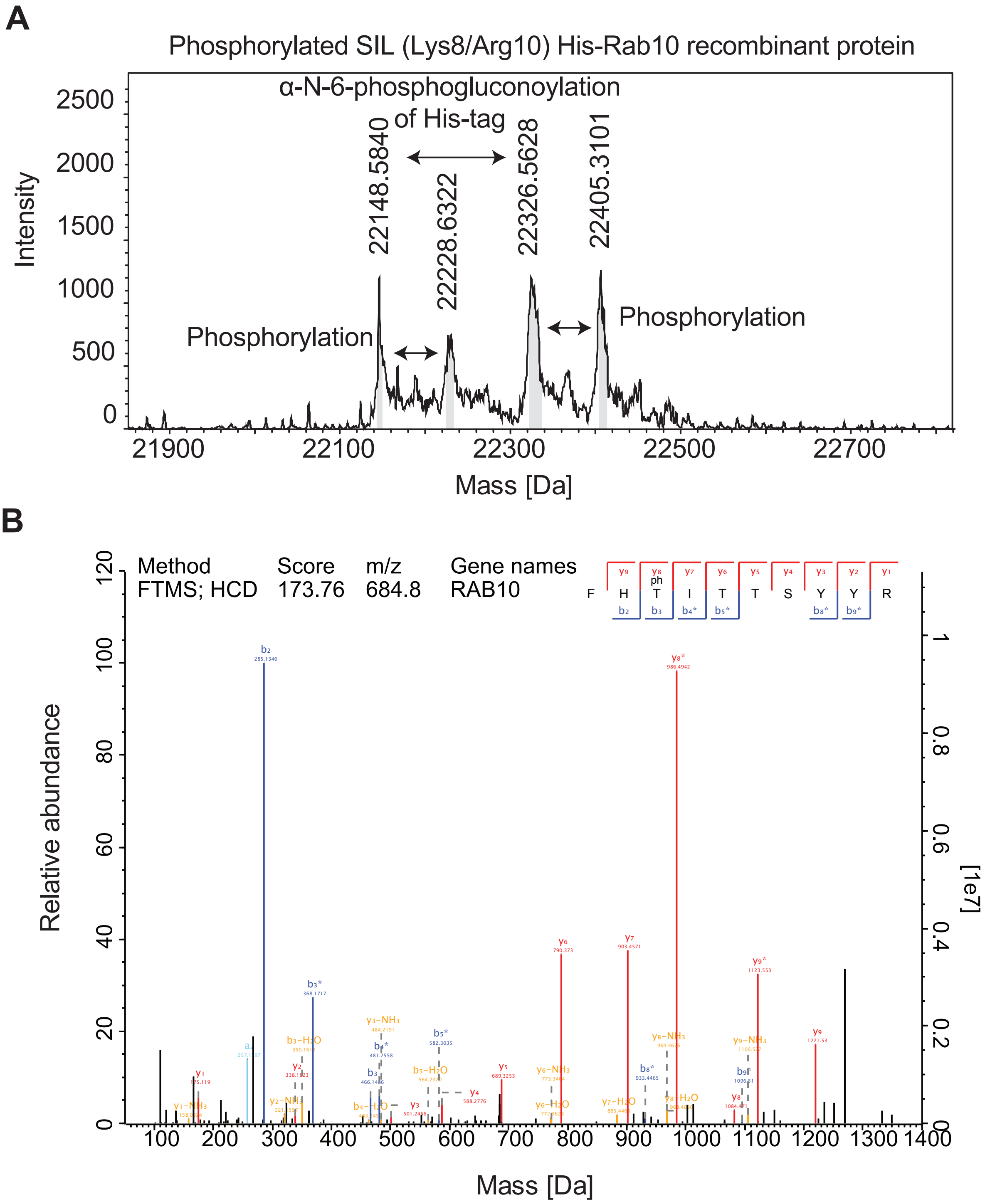

Supplementary Fig. 4. Phosphorylated SIL (Lys8/Arg10) His-Rab10 recombinant protein measured by both intact mass analysis and bottom-up proteomics. His-tagged Rab10 protein expressed and stable isotopically labeled (Lys8/Arg10) in E.coli was phosphorylated by the truncated (950-2527) recombinant human LRRK2-G2019S (Thermo Fisher, PV4881) in vitro. (A) The intact mass of the protein was analyzed by mTOF. We observed the mass of the protein (residues 1-175, m=22,148 Da) with a second peak with a mass difference of 178Da (m=22326 Da), indicative of α-N-6-phosphogluconoylation of His-tag (Geoghegan, K. F. et al., 1999) and 80 Da mass increases in both peaks due to phosphorylation. (B) LC-MS/MS analysis after digestion confirmed the phosphorylation at T73. The collision-induced dissociation (CID) fragmentation spectrum with the Andromeda score (Cox et al., 2011) are shown.

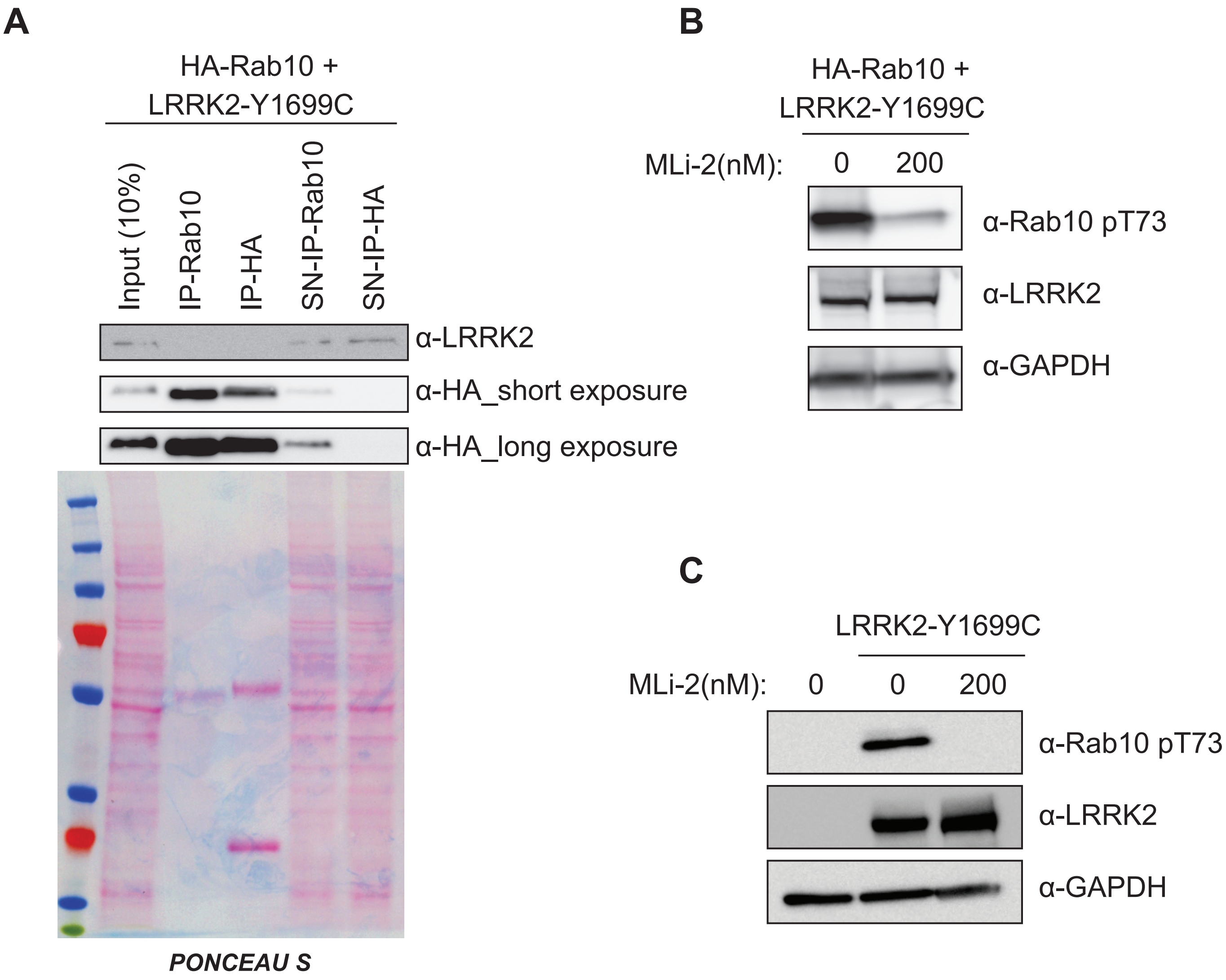

Supplementary Fig. 5. Rab10 protein either ectopically expressed or endogenously present in HEK293 cells with LRRK2-Y1699C expression. (A) Immunoblotting of HA-tagged Rab10 protein immunoprecipitation using HA agarose beads or anti-Rab10 antibody in HEK293 cells expressing LRRK2-Y1699C. The heavy chains can be seen in Ponceau staining. Input: whole cell lysate, IP: eluate, SN: supernatant. Immunoblottings of (B) HA-Rab10 and LRRK2-Y1699C and (C) mock and LRRK2-Y1699C expressing HEK293 cells (-/+ 200 nM MLi-2, 60 min) using total LRRK2, MJFF-pRAB10 (pThr73) and loading control GAPDH antibody.

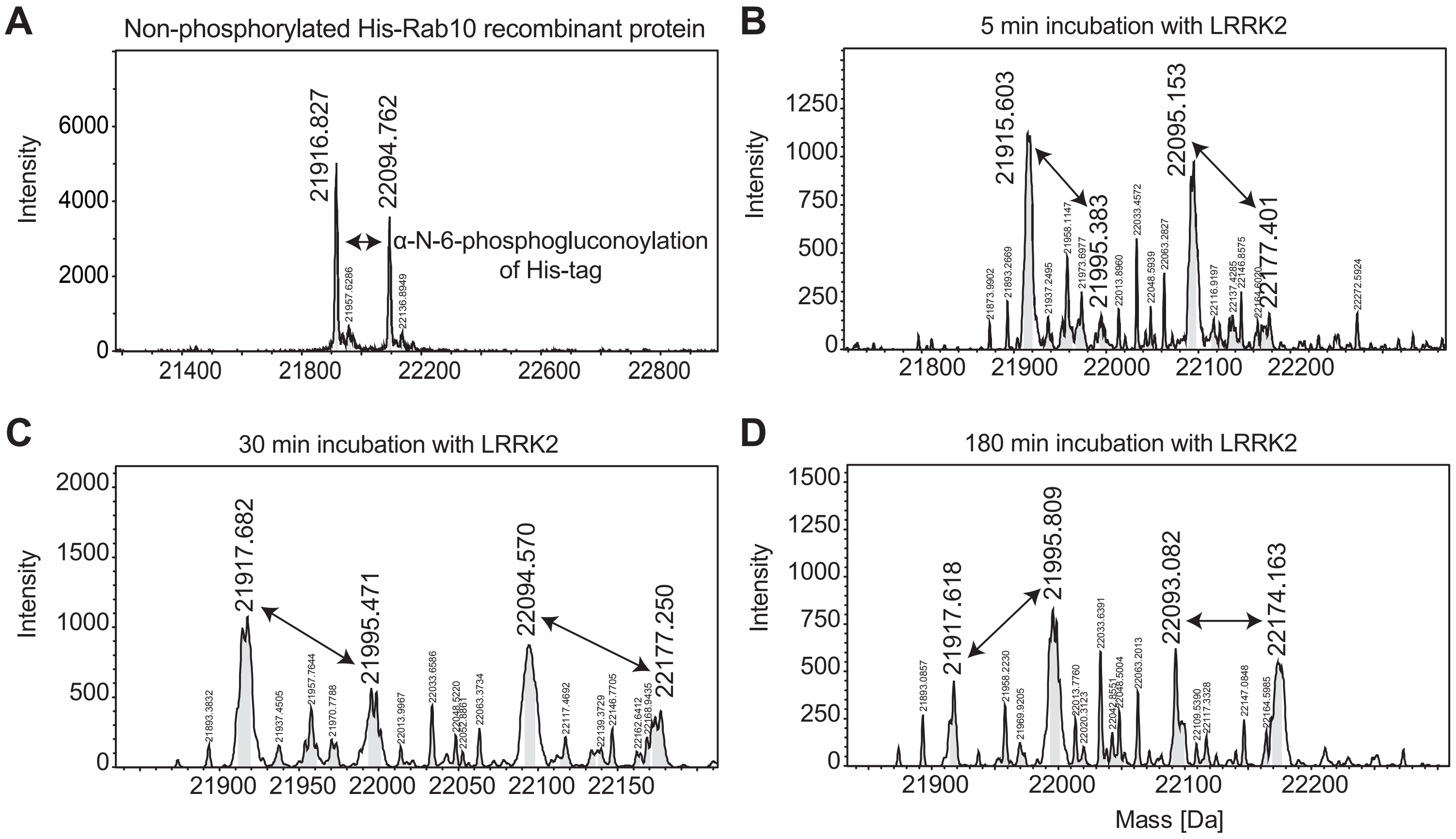

Supplementary Fig.6. Intact mass analysis of non-phopshorylated and phosphorylated unlabeled His-Rab10 recombinant protein. (A) Rab10 protein expressed in E.coli was analyzed by mTOF. We observed the actual mass of the protein (m=21,916 Da) and α-N-6-phosphogluconoylated version (m=22,094 Da) (Geoghegan, K. F. et al., 1999). It was phosphorylated by the truncated (950-2527) recombinant human LRRK2-G2019S (Thermo Fisher, PV4881) in vitro. The reaction was stopped by the addition of 2 µM of HG-10-102-0, a selective LRRK2 inhibitor, after (B) 5 min, (C) 30 min and (D) 180 min to obtain phosphoprotein standards with different occupancies. Intact mass analysis by mTOF confirmed the phosphorylation events by identification of 80Da mass shifts for both unmodified and α-N-6-phosphogluconoylated peaks.

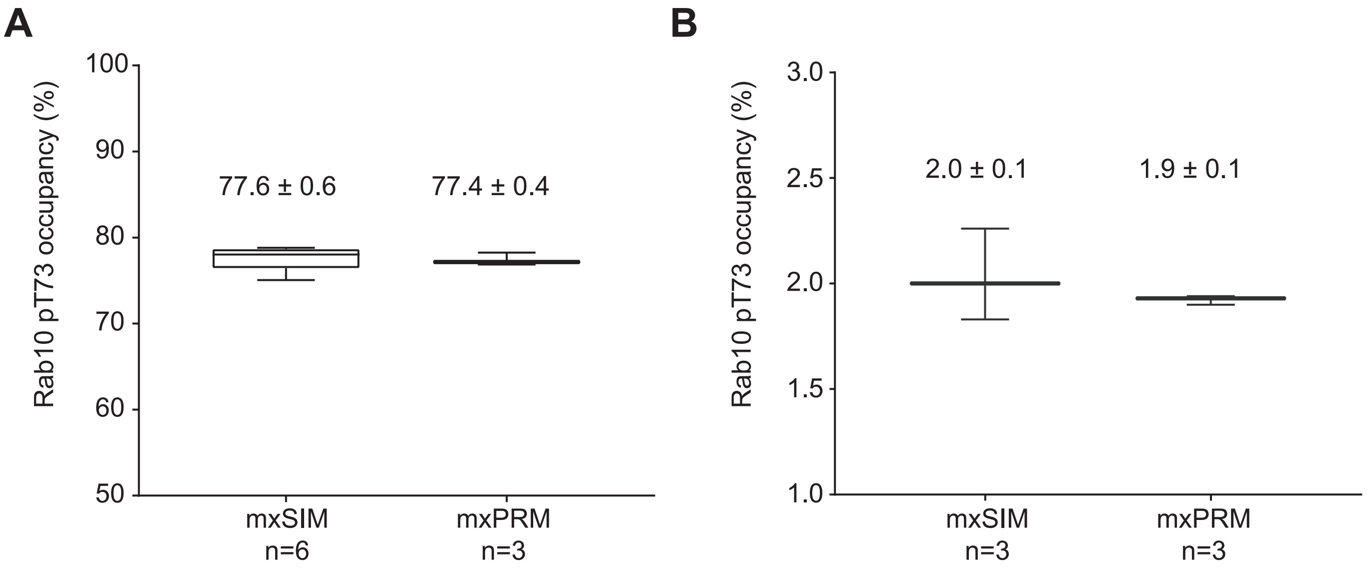

Supplementary Fig. 7. SIL phosphorylated and non-phosphorylated Rab10 peptides mixed in 50% occupancies and spiked into standard phosphoprotein digest (A) and neutrophils (B) were measured using either mxSIM method or mxPRM in replicates, respectively. The ccupancies were presented as means ± SEM.
